# Supplementary material for: Gradual positive and negative affect induction: The effect of verbalizing affective content
Source: PLoS One. 2020 May 29;15(5):e0233592. doi: 10.1371/journal.pone.0233592 (PMC7259663; doi:10.1371/journal.pone.0233592)
Supplement: S1 Table — (DOCX) [file pone.0233592.s001.docx]

**Table S1. Positive and negative IAPS pictures that were initially selected but excluded based on our exclusion criteria**

|  | Bin | Original | Reason exclusion | Replaced with |
| --- | --- | --- | --- | --- |
| Positive | 6 | 4559 | erotic or sexually suggestive | 7096 |
|  | 8 | 4275 | erotic or sexually suggestive, culturally sensitive | 2506 |
|  | 18 | 8600 | culturally sensitive | 8467 |
|  | 24 | 4609 | erotic or sexually suggestive | 2339 |
|  | 37 | 5982 | repetitive, 5870 is very similar and was already included in the set | 2165 |
| Negative | 34 | 2345.1 | too gruesome | 9435 |
