# Supplementary material for: Gradual positive and negative affect induction: The effect of verbalizing affective content
Source: PLoS One. 2020 May 29;15(5):e0233592. doi: 10.1371/journal.pone.0233592 (PMC7259663; doi:10.1371/journal.pone.0233592)
Supplement: S2 Table — (DOCX) [file pone.0233592.s002.docx]

**Table S2. Final selection of positive (increasing in valence), negative (decreasing in valence), and neutral IAPS pictures.**

|  | Positive | Negative | Neutral |
| --- | --- | --- | --- |
| 1 | 7053 | 7053 | 7053 |
| 2 | 7550 | 2102 | 2211 |
| 3 | 7026 | 7187 | 7160 |
| 4 | 7710 | 7000 | 7003 |
| 5 | 2372 | 7185 | 7235 |
| 6 | 7096 | 7009 | 2890 |
| 7 | 2980 | 7175 | 1645 |
| 8 | 2506 | 2190 | 5920 |
| 9 | 7192 | 2830 | 7014 |
| 10 | 7351 | 9468 | 7056 |
| 11 | 2616 | 7110 | 1122 |
| 12 | 4536 | 7031 | 7247 |
| 13 | 2594 | 2810 | 7207 |
| 14 | 2500 | 1080 | 8232 |
| 15 | 7515 | 7054 | 2397 |
| 16 | 2010 | 1200 | 7255 |
| 17 | 4542 | 9926 | 7004 |
| 18 | 8467 | 1930 | 2122 |
| 19 | 2302 | 1090 | 5510 |
| 20 | 8179 | 1280 | 7045 |
| 21 | 2358 | 1220 | 2102 |
| 22 | 7250 | 9373 | 7185 |
| 23 | 8040 | 2715 | 2377 |
| 24 | 2339 | 1274 | 2880 |
| 25 | 5870 | 9622 | 2514 |
| 26 | 1601 | 2455 | 7476 |
| 27 | 4700 | 9330 | 7020 |
| 28 | 2352 | 3212 | 7012 |
| 29 | 7508 | 9611 | 7170 |
| 30 | 4603 | 2750 | 7640 |
| 31 | 5001 | 9400 | 7077 |
| 32 | 8492 | 9050 | 6150 |
| 33 | 5270 | 9904 | 2038 |
| 34 | 2208 | 9435 | 7017 |
| 35 | 2274 | 3500 | 7002 |
| 36 | 2395 | 9254 | 2411 |
| 37 | 2165 | 2703 | 7043 |
| 38 | 2209 | 3350 | 7179 |
| 39 | 2530 | 9183 | 2749 |
| 40 | 5760 | 9410 | 2214 |
