# Supplementary material for: Gradual positive and negative affect induction: The effect of verbalizing affective content
Source: PLoS One. 2020 May 29;15(5):e0233592. doi: 10.1371/journal.pone.0233592 (PMC7259663; doi:10.1371/journal.pone.0233592)
Supplement: S3 Table — (DOCX) [file pone.0233592.s003.docx]

**Table S3. Self-referential and other-referencing in the pictures descriptions, sorted by condition, counted by LIWC**

|  | Selected words | Positive | Neutral | Negative | Total |
| --- | --- | --- | --- | --- | --- |
| Self-referential | ik zie  ik denk  het lijkt me  dit lijkt me  ik heb geen idee | 377 (23%) | 436 (28%) | 448 (28%) | 1261 (26.3%) |
| Other-referential | Hier zie je | 6 (0.4%) | 0 (0%) | 12 (0.8%) | 18 (0.4%) |
