# Supplementary figures and images for: Gradual positive and negative affect induction: The effect of verbalizing affective content
Source: PLoS One. 2020 May 29;15(5):e0233592. doi: 10.1371/journal.pone.0233592 (PMC7259663; doi:10.1371/journal.pone.0233592)

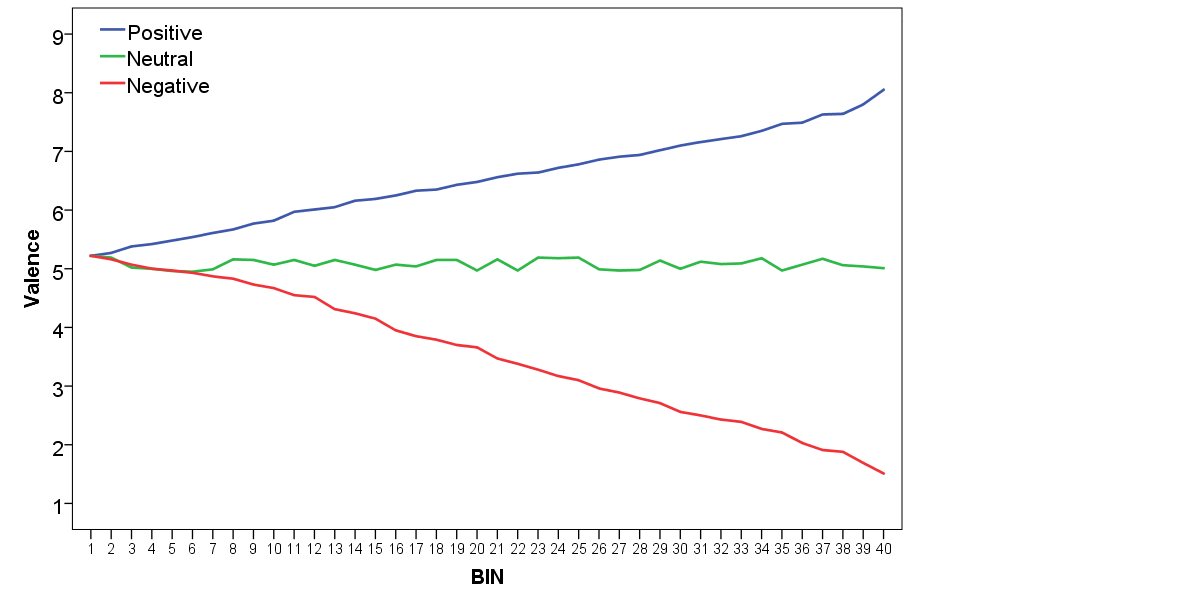

Supplement: S1 Fig — (TIF) [file pone.0233592.s004.tif]

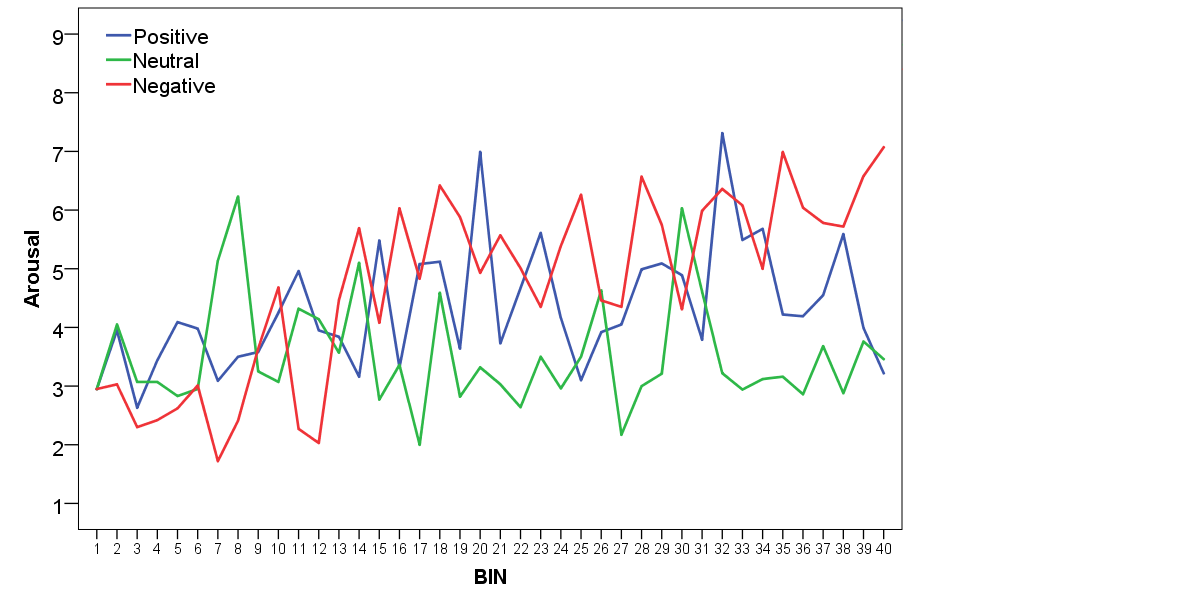

Supplement: S2 Fig — (TIF) [file pone.0233592.s005.tif]
