# Supplementary material for: Gradual positive and negative affect induction: The effect of verbalizing affective content
Source: PLoS One. 2020 May 29;15(5):e0233592. doi: 10.1371/journal.pone.0233592 (PMC7259663; doi:10.1371/journal.pone.0233592)
Supplement: Attachment — Submitted filename: Comments reviewers _revision.docx [file pone.0233592.s006.docx]

**Reviewer #1**

Review of Gradual positive and negative emotion induction using images: the effect of verbalizing emotional content (PONE-D-19-17931)

In this manuscript, the authors conducted two emotion induction experiments inspired by the Velten method. Using stimuli from IAPS, the authors placed participants in one of three emotion induction conditions: negative, positive, or neutral, where images either increased in negative valence, increased in positive valence, or remained neutral. In the first experiment, participants saw each image and described what they saw for 10 seconds in one of the three valence conditions. In the second experiment, participants passively viewed the images for 10 seconds each. The authors reported successful emotion induction, most successful for the negative condition. They found the induction results comparable to the Velten method.

The methods, analyses, and conclusions were sound. My main issue with the manuscript is the novelty of the question. To me this does not demonstrate a new method of emotion induction. Rather, it introduces additional complications that may not be necessary. One critical test of the method’s novelty would be to ask why an experimenter who wants to induce negative emotions would use this method rather than showing a random set of highly negative IAPS pictures?

*We thank the reviewer for their insightful and valid comments. We understand the main issue highlighted by the reviewer.*

*We realize now that we probably have not been clear enough in describing our aims and would like to clarify these below. The primary motivation was to study the effect of free verbal expression on emotion induction. Given that the relationship between language production and emotion induction is not well understood, we set out to investigate whether verbally describing emotional images would result in an enhanced emotional experience, compared to passively viewing the images. For this, we decided to pick a relatively natural and unconstrained approach, by presenting individuals with emotional IAPS images, asking them to describe the content freely.*

*To bring out this focus more clearly in the revised manuscript, we have adapted the title and we have now included a section of the paper exploring the content of participant’s verbal descriptions of the IAPS images. These descriptions were transcribed, and analyzed using Linguistic Inquiry and Word Count (LIWC), focusing on the production of emotional words as a function of the three conditions (positive, neutral, and negative), and over time (from the first image to the last image).We describe these in the Results section of a new subsection, ‘Exploratory analyses: Content analysis of image descriptions’.*

*A secondary motivation of our study is indeed exploring alternative emotion induction methods. As highlighted by, for example, Schmidt et al. (2018), most emotion induction methods are passive and not very ecologically valid. In our study, participants actively engaged in the induction method, especially in the setting where they were asked to verbally describe the images in such a way that other people would be able to identify the described image. Instead of reading out loud fixed statements (as in the Velten method), participants were asked to think for themselves and use their own words. We think this contributes to the need for more ecologically sound emotion induction methods. One attractive feature of the Velten method is its incremental nature. Somewhat surprisingly, this unique property of the procedure has not been studied or discussed in the literature, leaving us wondering whether the incremental manner of inducing mood is also effective when using (IAPS) images with affective content.*

*In the new manuscript, we now describe our focus more clearly in the Introduction.*

The gradual increasing of affective images might complicate emotion induction, such as imposing order effects and taking longer (400 seconds in this case). Alternatively, other studies have randomized blocks of negative images distributed throughout a task to remove order effects and to sustain negative affect induction. By using one long series of images, it is also possible that participants are just more responsive to the last portion of the most negative images, or that the most negative images at the end are made less salient because participants are slowly ramped up to them.

*Thank you for this comment. Indeed, for emotion induction purposes, it is common to present IAPS images in a random order (e.g., Capecelatro et al., 2013; Dhaka & Kashyap, 2017).*

*We conjecture that gradually increasing emotional valence of stimuli might have several advantages. First, the first stimulus is unlikely to shock or startle participants. When participants are startled by images, they might feel excessively tense and want to quit engaging in the study, which is both detrimental to the quality and quantity of the research, as well as ethically questionable. Velten argued in favor of gradual emotion induction, ‘to overcome the subjects’ presumable reluctance to experience unpleasant mood’ (Velten, 1967, p.68). Additionally, for some populations, the startle effect might be specifically unethical, because they could cause serious psychological or physiological harm, for example, individuals with certain mental disorders (e.g., PTSD, panic disorder), cardiovascular diseases, and the elderly.*

*Incidentally, we were unsure what the reviewer referred to with ‘taking 400 seconds longer’. We reckoned that they referred to the Spoken Descriptions of Images study, where we state that each image was viewed for 10 seconds, resulting in 400 seconds of viewing images (40 images x 10 seconds). We did not include information on the viewing time of the images in the Passively Viewing Images study, which might have been confusing. The viewing time per image was identical in both studies. We have now added this information to Passively Viewing Images study (Materials).*

Describing while viewing emotional images adds another layer of complication, such as attention and verbal skills. This may be more relevant as a comparison to other induction methods, or for explicitly testing the influence of verbalizing when exposed to emotional stimuli, which the authors partially reviewed but was not the intended question.

*First, we would like to clarify that we did intend to explicitly test the influence of verbalizing when exposed to emotional stimuli (as we described in response to the first comment from this reviewer). One of our initial hypotheses was that describing emotional images would enhance the effect on emotional state compared to passively viewing the images. We compared the two methods and concluded that there was no substantial difference in effect on mood.*

*We agree that attention and verbal skills could play a role in our ‘Describing and viewing images’ study. We did consider this, for example, by defining relevant exclusion criterion, such as having a speech disorder or limitation in the ability to speak fluently (for example, stuttering). Moreover, given that we tested a relatively homogenous group, we expect that other variabilities (cognitive and communicative abilities) will be randomly distributed throughout the sample, and therefore not have a major impact on our results.*

*We have reframed the revised manuscript, focusing more on the effect of verbalizing on affective state. We have added a new section, ‘Exploratory analyses: Content analysis of image descriptions’, exploring the content of the verbal descriptions of the images using LIWC, and also briefly discuss these results in the General Discussion.*

To clarify my issue, for example, if a gradual increasing method showed a more sustained or a different kind of negative affect induction, it would demonstrate novelty. Alternatively, if the authors could demonstrate specific behavioral consequences of this emotion induction method, or a particular reason for which a gradual increase of valence or the description of emotional stimuli is useful, or a particular set of questions or dependent measures that are differentially targeted by description of pictures rather than passive viewing, it would demonstrate novelty.

*We understand the reviewer’s point. As described above, in the revised manuscript we describe more explicitly that our primary interest is to study the effect of spoken language production on affective state. To demonstrate the novelty of our study more clearly, we have now transcribed and analyzed the verbal descriptions of the images using LIWC, which offers an additional dependent measure. Additionally, we have strengthened our discussion of the possible uses of our approach as an emotion induction method.*

Other notes:

What was the dependent variable of the ANOVAs? If it was valence ratings, using a magnitude change may reveal a greater and the interaction effect smaller. If it's not a magnitude change, if both negative and positive inductions were perfectly and equally effective, you would hypothesize a null time effect and predict only the interaction.

*In our analysis, we used a composite score (which we called "Affect") based on the 6 scales introduced in Mackie and Worth. This score ranges from 1 (very negative) to 7 (very positive) (see Materials for details). We have added this information now to ‘Change in emotional state' (both 2.2.2. and 3.2.2.). We agree with the reviewers interpretation of the possible effects; if both the negative and positive mood inductions were equally effective, we would get a null effect of mood induction (because the effects of the positive and negative condition cancel each other out) and only get an interaction between time and mood (because over time, the scores for the positive and the negative condition do start to diverge). However, as discussed in both Results sections (2.2 and 3.2), in our studies, only the negative mood induction was found to be successful.*

-the other component of the Velten method is the self-referential nature of the stimuli. There is now a literature on the impact of affective words when they influence the self vs. another. If the main thrust of the question is a direct comparison with the Velten method, this would ideally be addressed.

*The reviewer has a good point; indeed, our study and the Velten method differentiate with respect to self-referencing. Even though our main focus is on language production (as explained above), we do feel it is interesting to address this topic. We now briefly discuss recent work on this (such as Soares et al., 2019). When inspecting the picture descriptions, we find that only a small percentage (26%) is self-referential (e.g., ’I see…’), as opposed to many utterances in Velten’s original method. This might partially explain why our positive emotion induction was not effective. We now briefly touch upon this in the General Discussion (‘Future research’). For future research, it might be interesting to compare a self-referential condition (‘I see a happy couple’) to a non-self-referential condition of verbal image descriptions (‘This is a picture of a happy couple’). We have included this suggestion in the Future Research section.*

-despite comparable ratings, the positive valence IAPS pictures are sometimes considered to be not as significant as the negative valence pictures, possibly related to the greater ease in inducing negative emotions (which might account for the reduced positive induction). Also within the negative pictures, I’ve noticed the disgust images are more effective inducers than the fear pictures.

*We thank the reviewer for this clear observation. Indeed, this is common problem inducing positive emotions with various emotion induction procedures, and we refer to this problem in the General Discussion. We now briefly touch upon this issue regarding the effectivity of the positive valence images of the IAPS in specific.*

-Figure 3 should be bar plots. Line suggests an ordinal or continuous relationship between the three experiments

*The reviewer is, of course, correct. We have replaced Figure 3 with a bar plot.*

- the comparison of the emotion induction results with another study that used the Velten method could be placed in the Results section.

*We have moved* *the comparison with the Wilting study to ‘5. Comparing our results to the original Velten method’.*

**Reviewer #2**

This is a sound and well presented study. It builds on earlier work on the elicitation of changes in emotional states via linguistic stimuli, and presents the results of two experiments involving visual stimuli.

However, a limited amount of space is devoted to a discussion of the findings. I suggest that the authors include more explanation of the findings and more comments on their implications.

It should also be clarified why the use of images, as opposed to linguistic stimuli, is described as more 'natural'.

*We thank the reviewer for their compliments, and agree with their suggestions. We have expanded our General Discussion section, now explicitly discussing the limitations and strengths of our study. We discuss the possibility of order effects, the additional challenges of the verbalization of the stimuli, and how our ‘Describing Images’ study contributes to the need for more ecological and engaging mood induction procedures. We have rewritten the future research section, and added an implications section. We have also added new material, now dedicating a section of the paper to the analysis of the content of the verbal descriptions of the IAPS images (‘Exploratory analyses: content analysis of image descriptions’).*

*We also explain in somewhat more detail what we mean when we claim that our emotion induction method is more natural than the Velten method. This is because participants in our study were able to use their own words to describe affective stimuli, as opposed to the Velten method in which they read out loud fixed affective statements.*
