# Supplementary material for: Gradual positive and negative affect induction: The effect of verbalizing affective content
Source: PLoS One. 2020 May 29;15(5):e0233592. doi: 10.1371/journal.pone.0233592 (PMC7259663; doi:10.1371/journal.pone.0233592)
Supplement: Attachment — Submitted filename: Comments revision_2.docx [file pone.0233592.s007.docx]

Review of Gradual positive and negative emotion induction using images: the effect of verbalizing emotional content (PONE-D-19-17931)

**Reviewer #3**

This paper introduces new data that can be brought to bear on existing debates about the influence of language on (the intensity of) affective experience. The authors have made some valuable changes and additions in their revisions - in particular, the examination of the language used to describe the images. Yet there are still some points where clarifications and perhaps new analyses are needed. Please note that, although I did not serve as a reviewer during the first round, I read both versions of the manuscript before preparing my comments on the revision.

*We thank the reviewer for their comments and compliments.*

1. I would be cautious with the use of the word ‘emotion(al)’ throughout the manuscript. What is being induced and measured is a change in affect, valence, or un/pleasantness. The ‘affective content’ of the stimuli is therefore a more appropriate description. Likewise, I would refer to an ‘affect induction’ rather than an ‘emotion induction’, to ‘affective language’ rather than ‘emotional language’, etc.

*The reviewer is correct and we have changed this accordingly.*

2. At several points in the manuscript, the authors appear to conflate valence, arousal, and intensity (e.g. page 9 line 221). In relation to the selection of stimuli, I would also like to see the authors comment on how the “strong increase in arousal for negative images” (page 9, line 226) does or does not impact their results and conclusions. Could it be that this arousal difference is driving the negative affect induction? Relatedly, the authors suggest that positive and negative images may be more arousing than neutral images, which may explain differences in the number of words used in image descriptions (page 24, line 569). This conjecture could be directly tested.

*Regarding the conflation of valence, arousal and intensity, we have rewritten the manuscript, carefully considering the appropriate use of these terms. For example, we have changed line 221 to ‘affective content’ instead of ‘intensity’.*

*Indeed, the strong increase in arousal for negative images might impact our results and conclusions. To control for this, we followed the suggestion of the reviewer, now also looking at the correlations between the IAPS valence and arousal scores for the positive and negative images and the average number of words used by participants to describe these images. The results indicate that both valence and arousal were correlated to the number of words used to describe the images in the affective conditions*, *however, correlations between word use and valence were substantially higher.* *We describe these results in the Discussion.*

3. Can the authors clarify why they analyzed pre- and post-induction ratings separately, rather than creating change scores? It seems that change scores would preserve individual differences while streamlining the analyses and narrative. If the authors believe change scores are not appropriate, then I would highly recommend transforming the affect ratings to be centered on 0, such that negative ratings indicate negative affect, and positive ratings indicate positive affect. This will greatly assist in the interpretation of results and figures. For example, lines 276-8 (“lower scores indicate higher levels of negative emotion; higher scores indicate higher levels of positive emotion”) would be much easier to follow if scores were centered on 0.

*To some extent, this might to be a matter of subjective preference. Given that the effect of time (pre- and posttest) is important for answering our hypothesis (does affective state change after viewing/describing positive, neutral or negative images?), we prefer the repeated measures analyses keeping both the pre- and posttest scores, because we feel the results are more intuitive and easier to interpret than change scores. Change scores also make it harder to see how participants actually reported to feel in the different conditions.*

*However, we understand the concern of the reviewer and agree that the results sections could be improved to make them more easy to interpret. Therefore, we have now added change scores to Table 2, and clarified our explanations of the results by streamlining our language following the suggestions of the reviewer.*

4. There appears to be a slight mis-interpretation of the main effects reported on page 16 of the results. On lines 385-6, the authors state that “describing images enhanced emotional state in the participants”. To the contrary, a main effect of study type merely indicates that affect was more pleasant overall in Study 1 – this effect alone does not indicate an effect of the induction. Similarly, on lines 390-1, the authors state that “the emotion manipulation was effective for individuals exposed to negative images”. A main effect of condition simply means that affect was lower overall in the negative condition; causality cannot be inferred without examining change in affect.

*The reviewer is correct; thank you for spotting this. Regarding the first comment, we have changed this to “(…) indicating that affective state was overall more positive for participants in Study I, compared to Study II”. Regarding the second comment, we have changed this to “(…) indicating that affect was lower overall in the negative condition, both for participants*

*that described the images and for those that did not.’*

5. Much of the authors’ argument throughout the manuscript hinges on the effectiveness of having participants verbally describe the evocative images. Yet this argument isn’t always supported by the analyses and results. For example, in comparing the present studies with the work of Wilting et al. (pages 17-8), the authors only analyze the post-induction affect ratings for all 3 studies. Without taking pre-induction ratings into account, however, this comparison isn’t meaningful (i.e. without controlling for where participants started, we don’t know how much they were influenced by the task).

*Unfortunately, the pre-test scores of the Wilting et al. study were never collected. We understand the reviewer’s concern and have therefore decided to removed this comparison.*

Similarly, the authors state that “verbally describing emotional images is an effective method to induce, especially negative, emotional states” (page 22, lines 525-6), while the results indicate no effect of study (page 17, lines 401-2).

*We have rephrased this sentence to “Verbally describing and passively viewing affective pictures successfully induced negative affective states (in the negative condition), but not positive affective states (in the positive condition). Our findings did not support the hypothesis that verbally describing affective pictures would induce stronger affective states than passively viewing them.’*

Also, the claim that “participants generally reported stronger emotions after describing emotional images compared to only viewing them” (page 23, lines 533-4) conflates valence with intensity – higher ratings indicate more pleasant affect, which actually works against the effectiveness of a negative affect induction. These aspects should be revised to clarify the findings and contribution of the present work.

*We have changed this sentence to ‘Participants reported slightly more positive affect after describing the positive images, compared to passively viewing them. However, this difference was small and not significant.’*

6. Miscellaneous:
a. I prefer the original title, as the revised title seems to place undue emphasis on the effect of language on emotion (see above), and is also easy to misread as “verbalizing images gradually increases emotional content”
b. The original version of the manuscript included a brief description of the Velten method. This seems to have been removed in the revision, and I think it should be added back to help readers such as myself who were not otherwise familiar with the method.
c. Page 5, line 123: “especially for individuals who are free to use their own words” – I would rephrase as “especially when individuals are allowed to use their own words”
d. “Self-referral” (e.g. page 3, line 68) should be “self-referring” or “self-referential”
e. Page 9, line 212: what does it mean that the neutral images were pseudo-randomly selected?
f. Page 10, line 236: “electing” should be “eliciting”
g. Page 20 line 469: “Data were aggregated” (data is a plural noun)
h. Page 22, line 530: I would replace “happiness” with “positive affect”

*We have changed the title to “Gradual positive and negative affect induction by describing versus viewing affective pictures’’. Moreover, we have added a brief description of the original Velten method to the Introduction. The small errors have been corrected.*

*Regarding e), we understand the confusion and changed ‘pseudo-random’ to ‘random’, because neutral images were selected in a similar fashion as the affective images, i.e. at random.*

**Reviewer #4:**

The Authors did addressed all the points raised by reviewer 1. However, some of the authors' responses raise more questions. In particular, regarding the claims of incremental mood induction being "ecologically superior", the authors also pointed out that incremental induction of emotion has not been tested before on affective content images. Thus, only further investigations will support their statement of ecological validity. I suggest deleting lines 615-624 of the draft, and reference to "ecological sound" as a suggestion and not as a fact (lines 658-660 of the draft).

*We thank the reviewer for this observation and understand the need for clarification.*

*We have substantially rephrased lines 615-624, now suggesting that the engaging nature of the method is a potential strength, and we have removed mentioning of ecological superiority. In the Implications section of the Discussion, we now suggest that our method is possibibly more ecologically sound, instead of stating this as a fact.*
